# Effects of a bifid triple viable capsule on inflammatory factors and intestinal barrier function in children with abdominal infection

**DOI:** 10.1016/j.clinsp.2025.100731

**Published:** 2025-08-21

**Authors:** XiaoHui Yan, XiaoDan Zhong, XiaoXiao Zhang

**Affiliations:** a Department of Pediatrics, The People's Hospital of Danyang, Affiliated Danyang Hospital of Nantong University, Zhenjiang City, Jiangsu Province, China; b Guangzhou University of Chinese Medicine, Guangzhou City, Guangdong Province, China; c Department of Pediatrics, Chongqing Jiangbei Hospital of Traditional Chinese Medicine, Chongqing City, China

**Keywords:** Bifid triple viable capsule, Abdominal infection in children, Inflammatory factors, Intestinal mucosal barrier function

## Abstract

**Objective:**

To investigate the effects of Bifid Triple Viable (BTV) capsule on inflammatory factors and intestinal barrier function in children with abdominal infection.

**Methods:**

100 children with abdominal infections were selected. The control group (50 cases) was given conventional treatment, and the observation group (50 cases) was given BTV capsule treatment on the basis of the control group. The remission of clinical symptoms was compared, and the changes in inflammatory factors, intestinal mucosal barrier function indices, intestinal flora number, and immune function indices were observed, and the occurrence of adverse reactions during treatment was statistically analyzed.

**Results:**

The time of vomiting remission, fever remission, stool recovery, and abdominal pain remission in the observation group was shorter than those in the control group. IL-6, CRP, and TNF-α in the observation group were lower and IFN-γ was higher compared with the control group. DAO, D-lactic acid, LPS, Occludin, and ZO-1 in the observation group were lower than those in the control group. The quantity of *bifidobacterium, acidobacteria, and clostridium leptum* in the observation group was higher than that in the control group, and the quantity of *enteric bacillus* and *enterococcus* was lower. IgA and IgG levels in the observation group were lower than those in the control group after treatment.

**Conclusion:**

BTV capsule can reduce the local inflammatory response of abdominal infection and improve the intestinal mucosal barrier function and immune function.

## Highlights

• BTV capsule alleviates the local inflammatory response of abdominal infection.
• BTV capsule improves intestinal mucosal barrier function and immune function.
• BTV capsules effectively corrected the dysbiosis of gut microbiota.
• BTV capsule can reduce the IgA and IgG levels of patients.

## Introduction

Abdominal infection in children refers to intraperitoneal infectious diseases occurring in people under the age of 18, including intraperitoneal organ infection and internal and external cavity infection^1^^,^^2^. Clinically, abdominal infection may be caused by low immunity and intra-abdominal organ infection, trauma, and surgery. In addition, abdominal infection in children may lead to pathological inflammatory responses and aggravate the severity of the disease^3^^,^^4^. With the upgrading of diagnosis and treatment techniques and drugs related to abdominal infection, the clinical treatment effect of this disease has been greatly improved. Bifid triple viable (BTV) capsule is a microecological preparation composed of *bifidobacterium, lactobacillus*, and *enterococcus faecalis*, which can supplement the number of physiological flora in the intestine, correct the disturbance of intestinal flora, repair intestinal function, and thus alleviate clinical symptoms of patients^5^^,^^6^. Based on this, this study analyzed the effects of BTV capsule on inflammatory factors and intestinal mucosal barrier function in children with abdominal infection, aiming to explore the therapeutic effect of this drug in children with abdominal infection and provide references for the clinical treatment of this disease.

## Materials and methods

### *Clinical data*

100 children with abdominal infections from September 2020 to September 2021 were selected as the study objects and assigned to the control group and observation group by the random number table method, with 50 cases/group. The study was conducted with the informed consent of the patients and the approval of the Ethics Committee of The People's Hospital of Danyang (n° 202001DYPH-11). This study followed STROBE guidelines.

Inclusion criteria: 1) Met the diagnostic criteria for abdominal infection in children; 2) < 18-years-old; 3) The families of the children had signed the consent form.

Exclusion criteria: 1) Severe cardiac, liver, and renal dysfunction; 2) Secondary bacterial peritonitis; 3) Tuberculous peritonitis; 4) Malignant ascitis; 5) Other infectious diseases; 6) Previous history of abdominal surgery; 7) BTV capsule treatment before entering the group; 8) A history of drug allergy; 9) Combined immune system diseases; 10) Other intestinal diseases.

### *Treatment methods*

The control group was given targeted treatment according to the Expert consensus on diagnosing and treating intra-abdominal infections in Chinese children, and the observation group was given BTV capsule (210 mg/capsule, viable bacteria count not less than 1.0 × 10^7^ CFU, 420 mg (2 capsules)/times, 3 times/day, orally for 2 weeks) on top of the control group.

### *Outcome measures*

1) Relief of clinical symptoms: Vomiting remission time, fever remission time, stool recovery time, and abdominal pain remission time were compared. 2) Inflammatory factors: IL-6, CRP, TNF-α, and IFN-γ were detected by ELISA before and 2-weeks after treatment. 3) Intestinal mucosal barrier function indices: Fasting venous blood was collected before treatment and 2-weeks after treatment, and Diamine Oxidase (DAO) and d-lactic acid were detected by spectrophotometry. LPS, Occludin, and ZO-1 were detected by ELISA. 4) Intestinal flora: API20E microflora detection system was applied to detect the number of intestinal flora before treatment and 2-weeks after treatment. 5) Immune function: The immunoglobulin levels before and 2-weeks after treatment were detected by immunoturbidimetry using Dongzhi-40 biochemical analyzer. The kit was provided by Shanghai Fosun LONG March MEDICAL Science Co., Ltda.

### *Data analysis*

Sample size estimation referred to the “Calculation of Sample Size in Clinical Trials”, “Medical Statistics”, the sample size calculation formula was N=Z2×[P×(1−P)]/E2. At 95 % confidence level, *Z* was taken as 1.96, E was taken as 0.10, P was taken as 0.5, and it was calculated as *N* = 96, thus 100 cases were included in this study. All the data were processed using SPSS17 software. The statistical data were expressed as percentages and subjected to χ^2^ test. The measurement data were expressed as (± *s*) after a normality test and subjected to *t*-test; *p* < 0.05 meant that the difference was statistically significant.

## Results

### *Clinical data*

The clinical data showed no notable difference between the two groups (*p* > 0.05, Table 1).

**Table 1:** Comparison of clinical data between the two groups (*n* = 50).

Classification	Observation group	Control group	p-value
Gender			0.685
Male	31 (62.00 %)	28 (56.00 %)	
Female	19 (38.00 %)	22 (44.00 %)	
Age (years)	6.78 ± 1.13	6.91 ± 1.06	0.554
Disease course (days)	3.02 ± 0.58	3.07 ± 0.51	0.648
Infected bacteria			0.803
Gram-positive bacteria	9 (18.00 %)	11 (22.00 %)	
Gram-negative bacteria	41 (82.00 %)	39 (78.00 %)	
Intra-abdominal infection type			0.699
Suppurative appendicitis	21 (42.00 %)	17 (34.00 %)	
Primary peritonitis	16 (32.00 %)	19 (38.00 %)	
Intra-abdominal abscess	13 (26.00 %)	14 (28.00 %)	
Previous surgery	35 (70.00 %)	32 (64.00 %)	0.671
Antibiotic duration (days)	7.14 ± 2.11	7.68 ± 2.34	0.229
Antibiotic class			0.703
Carbapenems	6 (12.00 %)	8 (16.00 %)	
3rd-gen cephalosporin + metronidazole	31 (62.00 %)	27 (54.00 %)	
Piperacillin-tazobactam	13 (26.00 %)	15 (30.00 %)	

### *Clinical symptoms*

**Fig. 1:**
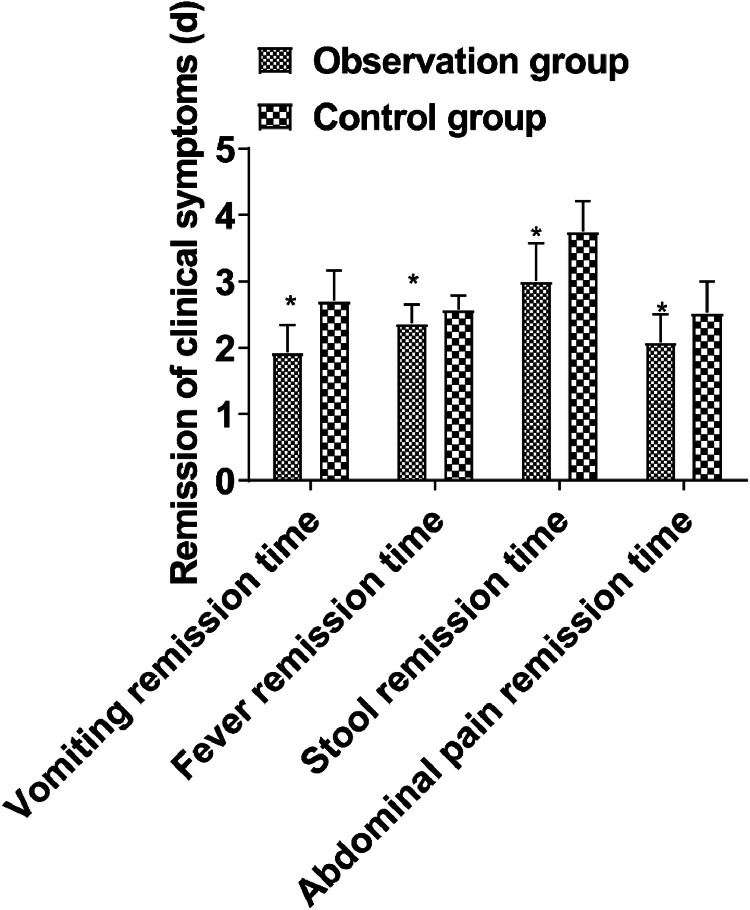
Comparison of clinical symptoms between groups. (Note: Compared with control group, * *p* < 0.05).

The time of vomiting remission, fever remission, stool recovery, and abdominal pain remission in the observation group was shorter than those in the control group (*p* < 0.05, Fig. 1).

### *Inflammatory factors*

**Fig. 2:**
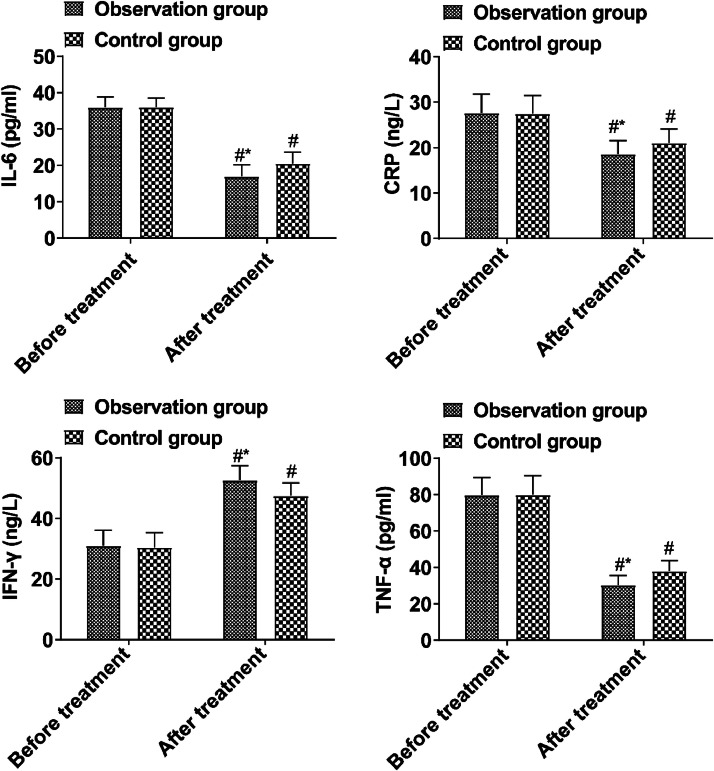
Comparison of inflammatory factors before and after treatment between the two groups. (Note: Compared with the group before treatment, # *p* < 0.05; Compared with control group, * *p* < 0.05).

Prior to treatment, the inflammatory factor levels in both groups showed no notable difference (*p* > 0.05). Post-treatment, the observed group's IL-6, CRP, and TNF-α levels were reduced compared to the control group, while IFN-γ levels exceeded those in the control group (*p* < 0.05, Fig. 2).

### *Intestinal barrier function*

**Fig. 3:**
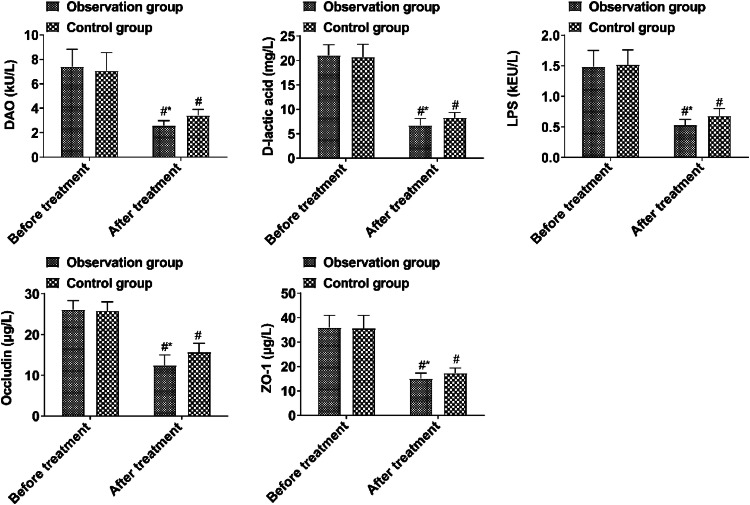
Comparison of intestinal mucosal barrier function indices between the two groups before and after treatment. (Note: Compared with the group before treatment, # *p* < 0.05; Compared with control group, * *p* < 0.05).

Prior to treatment, the intestinal barrier function showed no notable difference between the two groups (*p* > 0.05). After treatment, DAO, d-lactic acid, LPS, Occludin, and ZO-1 in the observation group were lower than those in the control group (*p* < 0.05, Fig. 3).

### *Intestinal flora*

No significant difference was observed in the number of intestinal flora between the two groups before treatment (*p* > 0.05). After treatment, the quantity of bifidobacterium, acidobacteria, and clostridium leptum in the observation group was higher than that in the control group, while the quantity of enteric bacillus and enterococcus was lower (*p* < 0.05, Table 2).

**Table 2:** Comparison of intestinal flora before and after treatment between the two groups (*10^7^ CFU, *n* = 50).

Groups	Time	Bifidobacterium	Lactobacillus	Clostridium leptum	Enteric bacillus	Enterococcus
Observation group	Before treatment	6.71 ± 1.03	3.79 ± 0.65	1.16 ± 0.18	4.03 ± 0.45	2.79 ± 0.32
	After treatment	11.45 ± 2.16^#^*	7.03 ± 0.51^#^*	2.31 ± 0.25^#^*	2.15 ± 0.39^#^*	1.28 ± 0.29^#^*
Control group	Before treatment	6.92 ± 1.12	3.85 ± 0.62	1.21 ± 0.22	4.16 ± 0.41	2.85 ± 0.39
	After treatment	9.86 ± 2.03^#^	5.82 ± 0.59^#^	1.95 ± 0.20^#^	2.57 ± 0.43^#^	1.60 ± 0.25^#^

### *Immune function*

**Fig. 4:**
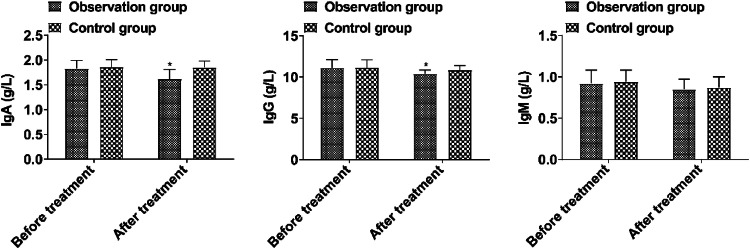
Comparison of immune function before and after treatment between the two groups. (Note: Compared with the group before treatment, # *p* < 0.05; Compared with control group, * *p* < 0.05).

Prior to treatment, the immune function index showed no notable difference between the two groups (*p* > 0.05). IgA and IgG in the observation group were lower than those in the control group after treatment (*p* < 0.05, Fig. 4).

### *Adverse reactions*

No obvious adverse reactions occurred in both groups during treatment.

## Discussion

Abdominal infection is a common and serious infectious disease in pediatric surgery, which can easily cause systemic inflammatory response syndrome. The treatment of children with abdominal infections with microbial preparations on the basis of antibacterial drugs can effectively improve the therapeutic effect^7^. BTV capsule is a microecological preparation commonly used in clinical practice. It contains bifidobacteria, *Lactobacillus acidophilus*, and enterococcus faecalis, which can effectively regulate the balance of intestinal flora and reduce the synthesis and secretion of intestinal endotoxin. In addition, bifidobacteria, L. *acidophilus*, enterococcus faecalis, and other live bacteria can completely cover the surface of intestinal mucosal tissue with other intestinal anaerobic bacteria. Thus, a biological barrier protection system is formed, which has an antagonistic effect on pathogenic bacteria by absorbing a large amount of nutrients^8^^,^^9^. In this study, it was found that the remission time of vomiting and fever, stool recovery time, and abdominal pain remission time in the observation group were shorter than those in the control group, suggesting that the drug can effectively reduce the clinical symptoms of children. The reason is that the BTV capsule can also acidify the intestine, help the rapid absorption of vitamin D, iron and calcium, improve the supplement efficiency of trace elements in the body of children, and promote the physical rehabilitation of children.

The balance of intestinal flora is closely related to the integrity of the mucosal barrier, which includes the biological barrier formed by the combination of intestinal dominant flora and intestinal mucous epithelial cells and the mechanical barrier formed by the tight connection between intestinal mucosal epithelial cells^10^. The disturbance of intestinal flora can directly affect the integrity of the intestinal mucosal biological barrier and cause bacterial translocation. The continuous proliferation of pathogenic bacteria can destroy the tight connection between intestinal mucosal epithelial cells through their metabolites, and then cause the destruction of the mechanical barrier of the intestinal mucosa. DAO is a metabolic enzyme in intestinal mucosal epithelial cells, and cell damage will cause DAO release into the blood circulation. LPS and d-lactic acid are the products of bacterial metabolism. The intestinal flora translocated into the blood circulation and peritoneal cavity secrete a large amount of LPS and d-lactic acid during metabolism. The integrity of the intestinal mucosal barrier can be reflected by measuring LPS and d-lactic acid in serum. Occludin and ZO-1 are molecules involved in the regulation of tight intercellular junctions. When tight intercellular junctions break down, Occludin and ZO-1 fall off and enter the blood circulation^11^^,^^12^. In this study, it was found that DAO, d-lactic acid, LPS, Occludin, and ZO-1 levels in the observation group were lower than those in the control group after treatment, indicating that BTV capsule could effectively improve intestinal mucosal barrier function. The reason is that the active ingredients in the BTV capsule are bifidobacterium, Lactobacillus, and enterococcus faecalis, which can correct the disturbance of intestinal flora and bind closely with intestinal mucosal epithelial cells to form a biological barrier after oral administration, thus inhibiting the translocation of intestinal flora to blood circulation and ascites. While reducing the translocation of intestinal flora into ascites, it is also conducive to the killing of original pathogens in ascites by antibiotics, thus improving the function of the intestinal mucosa^13^^,^^14^.

Intestinal microecological disorder can induce local inflammation and destroy the intestinal mucosal barrier function. Abnormal intestinal mucosal permeability will lead to intestinal bacteria producing a large amount of d-lactic acid and entering the blood circulation through the portal vein. In addition, when intestinal mucosal epithelial cells are damaged, DOA synthesis and release will be promoted and enter the blood. When the intestinal flora is picky, it can also lead to the proliferation of pathogenic bacteria in the intestine and the release endotoxins, further destroying the intestinal mucosal barrier. IL-6, TNF-α and CRP are all pro-inflammatory factors, and their levels are positively correlated with the degree of inflammatory response^15^^,^^16^. Intestinal mucosal injury can promote the release of a large number of local pro-inflammatory factors, triggering inflammatory responses. The results showed that IL-6, CRP, and TNF-α in the observation group after treatment were lower than those in the control group, and the levels of IFN-γ were higher than those in the control group, suggesting that BTV capsule could reduce the inflammatory response of children, which was mainly related to the improvement of intestinal mucosal barrier function by this drug.

Damage to the intestinal mucosal barrier function, disturbance of intestinal flora and translocation into blood circulation and ascites are pathological links of abdominal infection in children^17^. Anaerobic bacteria such as bifidobacterium, lactobacillus, and Clostridium teniculatum are the dominant bacterial groups in the intestine under physiological conditions, which can regulate the physiological function of the intestine and inhibit the reproduction of pathogenic bacterial groups. enteric bacillus and enterococcus are common pathogenic bacteria in the intestine, which can aggravate the further progression of the disease. BTV capsule can effectively supplement the missing physiological flora in the intestine, correct the disturbance of the flora, restore the homeostasis of the intestinal microenvironment, and form a biological barrier in the intestinal mucosa to prevent the continued invasion of pathogens, repair the damaged intestinal mucosal tissue, and inhibit the inflammatory response^18^^,^^19^. In this study, the number of bifidobacterium, acidobacteria, and clostridium leptum in the observation group after treatment was more than that in the control group, while the number of enteric bacillus and enterococcus was less, indicating that BTV capsule could effectively correct the intestinal flora disorder, mainly because BTV capsule is an intestinal microecological preparation, which can effectively correct the dysfunctional intestinal flora and repair the immune mechanism.

The immune and digestive systems of children are significantly different from those of adults, and the immaturity of the immune system leads to the inability of children to initiate a normal response when they suffer from abdominal infection. Bifidobacteria can activate immune cells in the intestinal mucosa and enhance the local defense function of the intestinal mucosa^20^. The results showed that the IgA and IgG levels in the observation group were lower than those in the control group after treatment, indicating that the drug can improve the immune function of patients. Despite the theoretical potential of the BTV capsule to improve the levels of inflammatory factors and intestinal barrier function in children with abdominal cavity infections, their practical application effects are limited by individual differences, the complexity of the mechanism of action, insufficient evidence from clinical studies, uncertainty of treatment timing and dosage, and interaction with antibiotics. Future studies need to further explore the individualized application strategies of probiotic preparations and optimize their therapeutic regimens in pediatric patients with abdominal infections, with the aim of improving clinical efficacy and safety. Meanwhile, it is important to strengthen basic research to understand the interaction of probiotics with the host immune system and gut microbiota to guide the rational application of probiotic preparations.

In summary, the treatment of BTV capsule alleviates the local inflammatory response of abdominal infection and improves intestinal mucosal barrier function and immune function.

## Data available

Data is available from the corresponding author on request.

## Ethics statement

All procedures performed in this study involving human participants were in accordance with the ethical standards of the institutional and/or national research committee and with the 1964 Helsinki Declaration and its later amendments or comparable ethical standards. All subjects were approved by The People's Hospital of Danyang (n° 202001DYPH-11).

## Funding

Not applicable.

## CRediT authorship contribution statement

**XiaoHui Yan:** Conceptualization, Methodology, Writing – original draft. **XiaoDan Zhong:** Formal analysis, Investigation, Visualization. **XiaoXiao Zhang:** Validation, Writing – review & editing, Supervision, Project administration.

## Conflicts of interest

The authors declare no conflicts of interest.
